# Microglia M2A Polarization as Potential Link between Food Allergy and Autism Spectrum Disorders

**DOI:** 10.3390/ph10040095

**Published:** 2017-12-09

**Authors:** Hans O. Kalkman, Dominik Feuerbach

**Affiliations:** 1 Gänsbühlgartenweg 7, CH4132 Muttenz, Switzerland; 2 Novartis Pharma AG, CH4002 Basel, Switzerland; dominik.feuerbach@novartis.com

**Keywords:** mast cell, Th2 cell, interleukin 4, insulin-like growth factor, autophagy, synaptic pruning

## Abstract

Atopic diseases are frequently co-morbid with autism spectrum disorders (ASD). Allergic responses are associated with an activation of mast cells, innate lymphoid cells, and Th2 cells. These cells produce type-2 cytokines (IL4 and IL13), which stimulate microglia and macrophages to adopt a phenotype referred to as ‘alternative activation’ or ‘M2A’. M2A-polarized macrophages and microglia play a physiological role in tissue repair by secreting growth factors such as brain-derived neurotrophic factor (BDNF) and insulin-like growth factor-1. In ASD there is evidence for increased type-2 cytokines, microglia activation, M2A polarization, and increased levels of growth factors. In neurons, these growth factors drive a signal transduction pathway that leads to activation of the enzyme mammalian Target of Rapamycin (mTOR), and thereby to the inhibition of autophagy. Activation of mTOR is an effect that is also common to several of the genetic forms of autism. In the central nervous system, redundant synapses are removed via an autophagic process. Activation of mTOR would diminish the pruning of redundant synapses, which in the context of ASD is likely to be undesired. Based on this line of reasoning, atopic diseases like food allergy, eczema or asthma would represent risk factors for autism spectrum disorders.

## 1. Introduction

Autism spectrum disorders (ASD) are a collection of neurodevelopmental conditions characterized by language deficits, social impairments, and repetitive behaviors [1,2]. These symptoms become apparent usually around 2–3 years of age and sometimes manifest with a regression in acquired language and behavioral skills. There are wide variations in clinical presentation and disease progression. Attempts to categorize ASD have resulted in the recognition of distinct subgroups: classic autism (which can entail general intellectual disability and language delay), Asperger syndrome (in which there is no language delay or intellectual disability), and several syndromic forms, in which high-impact gene mutations (e.g., Rett syndrome, Timothy syndrome, Fragile X or tuberous sclerosis) are important contributing factors. Cognitively, ASD is characterized by weakened executive function, reduced central coherence, and poorer mentalizing properties. Conversely, ASD patients may also have special ‘strengths’ (savant syndrome; [3]), such as attention to detail or systemizing (reviewed in [4]). 

Data from multiple studies indicate that the synapse may play a central role in the pathology of ASD [5,6,7,8,9,10]. Pruning of synapses and spines by microglial cells [11] is critically dependent on intraneuronal autophagy [12,13]. Stimulation of mTOR (mammalian target of rapamycin) displays an inhibitory effect on autophagy [13], whereas multiple ASD syndromes are caused by disruptive mutations in genes that, if intact, function as inhibitors of mTOR (for recent reviews see [14,15]). 

Whereas prevalence studies in twins with ASD indicate that the genetic factors play a dominant role in ASD prevalence [16,17], these studies similarly emphasize the importance of a shared environment [18,19]. Childhood disorders of the immune system (e.g., asthma, life-threatening food allergies) have reached “an epidemic level” over the past two decades [20] and the same is true for the ASD prevalence. As summarized by Estes and McAllister [20], in the United States the estimate in 1992 was 1 in 500 children, in 2007 this was 1 in 110, and recent estimates are 1 in 68 (*n.b.* 1 in 42 boys!). This drastic rise over such a short period of time cannot be explained by genetic changes and suggests that, apart from trivial reasons like increased awareness and diagnostic criteria that are more inclusive, recent environmental changes must contribute [20]. Unfortunately, the nature of environmental influences that are relevant for ASD remains largely undefined, although parallels have been drawn to the increasing prevalence of atopic disorders in the general population [21]. Food allergy in particular seems to be frequently comorbid with ASD [22,23]. In the present review, the hypothesis is put forward that allergies exacerbate ASD by an immunological mechanism that leads to activation of microglia. As a consequence, mTOR in neurons is turned on, leading to reduction of synaptic pruning. 

## 2. Environmental Risk Factors for ASD

In an epidemiological study, a doubled risk of ASD was found when mothers were diagnosed with asthma or allergy during the second trimester of pregnancy [24]. Maternal stress has been proposed as a factor that could lead to allergy or asthma [25,26,27] and ASD [28,29]. Becker [21] has reviewed the epidemiological, morphometric, molecular and genetic similarities between autism and allergic disorders, and suggested that the increase in ASD-prevalence might relate to the increase in atopic disorders owing to improvement in hygiene. Over the years, numerous studies have appeared that point to an association between ASD and atopic disorders. For instance, in a small study of individuals with Asperger syndrome, two thirds of the individuals suffered from atopic diseases (dermatitis, asthma or rhinitis) and had high serum levels of IgE and eosinophils [30]. In a longitudinal study of children with ASD and a control group of typically developing children, food, environmental and seasonal allergies were present in a minority only, but were significantly more frequent in the cohort with ASD compared to controls [31]. Angelidou et al. [32] proposed that mast cell activation could be an important pathological factor in ASD. These authors suggest that mast cell activation by allergic, infectious, environmental and stress-related triggers might release pro-inflammatory and neurotoxic molecules that disrupt the gut–blood, and blood–brain barriers, thus contributing to brain inflammation and ASD pathogenesis [32]. Head size of ASD patients at birth is often smaller, but in the period between approximately 2 months of age until approximately the 12th month of age, a growth spurt can result in abnormally large head sizes [33,34,35,36]. Interestingly, the presence of asthma, hay fever or high serum levels of IgE in pregnant mothers were prospectively associated with an increased head circumference in the offspring [37,38,39]. In an investigation of children diagnosed with allergy (asthma, atopic dermatitis, food allergy, high IgE), the head sizes at mid-gestational age (determined in utero by an ultrasound scan) were significantly smaller than non-allergic controls [40]. Thus, the data collected in allergic children resemble those from autistic children: a small head size in utero is postnatally followed by an overshooting growth spurt. In contrast to the association between food allergy and ASD [22,23], the association between respiratory allergy and ASD did not reach significance [23,41], but nevertheless respiratory allergy seems to exacerbate the behavioral symptoms of ASD [23].

## 3. Mast Cells, Th2 Lymphocytes, and Cytokine Profiles in ASD

The role of immune factors in ASD has been summarized recently in several excellent reviews [20,42,43]. After cross-linking of the cell-bound IgE-FcεRI complex, human mast cells release inflammatory mediators such as histamine and eicosanoids. In addition, mast cells are the sources of several cytokines like pro-inflammatory interleukins (IL1β, TNFα and IL6), interleukins related to tissue repair and allergy (IL4, IL5 and IL13), chemokines (CXCL8 and CCL2) and the growth factor VEGF [44,45]. Elevated concentrations of IL4 and IL5 were found in a study of banked serum collected from women during the 15th and the 19th week of gestation who gave birth to children who were ultimately diagnosed with ASD [46]. Furthermore, elevated levels of IL4, IL10 and TNFα were measured in the amnion fluid of fetuses that later developed ASD [47]. This small summary shows that elevated levels of mast cell products have been observed in *mothers* of ASD patients. As the immune system continues to mature in the postnatal period, one would also expect evidence for differences in the immune system of children with ASD compared to control children. Indeed, in neonatal blood spots collected from ASD patients and normally developing control children, the presence of elevated concentrations of IL1β and IL4 were independently associated with ASD [48]. This study noted that whereas IL1β was associated with increased odd ratios of mild/moderate ASD, an elevated level of IL4 was associated with severe ASD. Furthermore, IL4 levels were inversely correlated with nonverbal cognitive ability in a group of male ASD subjects. The authors thus concluded that peripheral cytokine profiles at birth are not only associated with ASD later in childhood, but also differ in relation to symptom severity [48]. In a further study, blood serum from adults with Asperger syndrome and controls was investigated [49]. In this study, male and female individuals with Asperger syndrome displayed very different cytokine profiles, with males expressing increased amounts of IL4, IL5, IL10, TNFα and a few other cytokines, while females mainly expressed altered amounts of growth factors (e.g., insulin, brain derived neurotrophic factor [both increased] and growth hormone and endothelin [both decreased]). Notably, there was very little overlap between the factors altered in male and in female Asperger individuals [49]. Several other studies have investigated cytokine profiles in blood and blood cells of ASD patients. An early study by Gupta et al. [50] found increases in typical Th1 cytokines (IL2, IL6 and IFNγ), as well as typical Th2 cytokines (IL4 and IL10) and an increase in the number of Th2-cells. This result is in agreement with data from Molloy et al. [51], but somewhat at variance with data from Suzuki et al. [52]. Molloy et al. [51] investigated peripheral blood mononuclear cells (PBMCs) from children with ASD and found that these cells produced higher amounts of Th2 cytokines (IL4, IL5, IL10 and IL13) than PBMCs from matching control subjects. Th1 cytokines were only slightly elevated. In contrast, Suzuki et al. [52] noted that Th1 and Th17 cytokines (IL1β, IL8, IL12p70 and IL17) in blood plasma of male high-functioning ASD patients were significantly elevated, whereas IL4, IL5 and IL13 were increased only trend-wise. Also Careaga and colleagues [53] investigated PBMCs of male ASD patients. Upon phytohemagglutinin-challenge, different subgroups were distinguishable: a group with an increased Th2 profile (increase in IL13), a group with an increased Th1 profile (IFNγ increase), and a third group with no typical response. In contrast to the third group, patients in the Th1 and Th2 groups were developmentally impaired [53]. To complete this summary, also a study by Hu et al. [54] provided evidence for a distinct Th2-related immune phenotype within ASD. These investigators generated lymphoblastoid cell lines from ASD-, and/or language-discordant monozygotic twins. Differences in gene expression were studied by microarray, and the data were used to establish networks of similarly regulated genes. One of the identified networks included the Th2-genes, IL5RA, IL13 and IRF4 [54]. The conclusion from these cross-sectional studies is that maternal and neonatal immune factors related to mast cells and Th2-skewed lymphocytes could play a role in increasing the risk of ASD. 

Quite recently, the first longitudinal study has become available, which did not substantiate the conclusion of the former cross-sectional studies. This study enrolled 104 children with ASD. The levels of 20 cytokines and 10 chemokines were assessed in serum but did not differ from the levels in an age-matched control group of 54 children. Moreover, the serum levels of analytes were poor predictors of the levels in the cerebrospinal fluid (CSF) [31]. This negative result conflicts with previous data, including those of the same research group [55]. Possible explanations involve a different compartment (brain tissue versus CSF), different assay techniques (multiplex bead assay versus immunostaining, Elisa or protein array) or a considerable temporal fluctuation in inflammatory process. 

## 4. IL4 Induces M2A Polarization of Macrophages and Microglia

Cells belonging to the monocyte-macrophage lineage have long been recognized to be heterogeneous. The apparent diversity of macrophages is most likely a functional adaptation to cues in the microenvironment. Similar to the Th1 and Th2 polarization of the adaptive immune system, a nomenclature has been proposed for the polarization of macrophages, with “M1” reflecting a distinct activation program seen in response to bacterial and viral infections, Toll-like receptor agonists, and pro-inflammatory cytokines (IFNγ, IL1β, TNFα or IL6). A distinctly different activation program that takes place in response to parasites, during tissue repair and in response to the cytokines IL4, IL5 and IL13 has been termed “M2” [56]. Mantovani and co-authors [56] distinguish several M2 subtypes (M2A, M2B and M2C); however, this subdivision is not generally accepted. Notably, very similar phenotypic changes have been observed in microglia, and the M1/M2 nomenclature is used for microglia polarization states as well [57].

IL4 is produced by mast cells, by Th2 and T_REG_ T-cells, as well as by innate lymphoid cells [44,58,59]. In B-lymphocytes, IL4 promotes immunoglobulin isotype-switching from IgG to IgE, and thereby contributes to the typical immune responses seen in allergy and asthma [60,61]. As an additional important activity, IL4 stimulates the differentiation of macrophages and microglia towards the M2A-phenotype (known as M2A “polarization”; see [58,62]). Similar to IL4, also IL13 is able to induce M2A polarization [63,64]. Whereas IL4 activates the IL4Rα receptor, IL13 activates the IL13Rα1 receptor. The two receptors can form heterodimers, which are responsive to both IL4 and IL13 [63]. IL5 activates IL5Rα receptor and this plays a role in eosinophilia [63]. The induction of an M2A phenotype by exposure to IL4 or IL13 provokes a near loss of the specific M1 marker, inducible nitric oxide synthase ‘iNOS’ [64]. Typical markers for M2A polarized macrophages and microglia are the arginase enzyme Arg1, scavenger receptors, and the mannose receptor [56,57,64]. The transcription of growth factors like VEGF, BDNF and PDGF is increased in IL4-induced M2A microglia [64]. In the M2A-polarized state, microglia cells also express insulin-like growth factor-1 [57,65]. As a consequence, allergy may indirectly lead to an increased release of a number of potent growth factors from microglia.

## 5. Changes in Post Mortem Brain of ASD

Direct evidence for microglia activation in the brain of ASD patients has been obtained with PET-radiotracer [^11^C](R)-PK11195 [66]. The tracer selectively labels the 18 kDa translocator protein (TSPO) in microglia. In activated microglia the expression of TSPO is strongly increased. The study by Suzuki and colleagues investigated adult male ASD patients and compared these to age- and IQ-matched controls. The binding values of the PET-ligand were significantly higher in multiple brain regions in ASD cases [66]. These results by Suzuki et al. [66] are complemented by a large longitudinal study in children with ASD by Pardo and colleagues [31]. Using repeated CSF sampling at different time points, it was noted that CSF levels of FLT3L, IL15, CX3CL1, CXCL8 and CCL2 were elevated [31]. Each of these immune mediators exerts a modulatory role on microglia and neuroglia–neuronal interactions. E.g., they promote cell differentiation, proliferation, and survival of microglia but also facilitate migration to areas of injury or play critical roles in the homeostatic function of microglia (see [31] for further details).

These in-life studies are further complemented by several post mortem studies. For instance, Morgan et al. [67] compared microglia in sections from the dorsolateral prefrontal cortex of 13 male ASD cases with that from control cases. The morphological alterations observed in ASD sections included somal enlargement, process retraction and thickening, and extension of filopodia from processes. Average microglial soma volume was significantly increased in white matter. These signs of microglia activation were seen in 5 out of 13 cases with ASD, whilst the effect was evident already at young age, since two cases were less than 6 years old [67]. Whereas such data provide evidence for microglia activation in ASD and corroborate well the TSPO study by Suzuki [66] mentioned above, they do not allow a distinction between the classical ‘infection-related’ M1-phenotype or the allergy and repair-related M2A phenotype [58]. Measurements of cytokine levels and microglia markers provide clear evidence for M1 polarization in frontal cortex samples. A study by Li et al. [68] found increased levels of TNFα, IL6, GM-CSF, IFNγ (Th1 cytokines), whereas IL4, IL5 and IL10 were not altered. However, the majority of post mortem studies in ASD suggest a mix of M1 and M2 states [55,69,70]. During infections, M1-polarized microglia produce several cytotoxic molecules, such as quinolinic acid, as well as oxygen and nitrogen radicals [71,72,73,74]. The radicals oxidize tetrahydrobiopterin (also known as BH4; an important cofactor for the biosynthesis of monoamines) to the functionally inactive metabolite neopterin [75]. In a remarkable study, Zimmerman et al. [76] noted that the levels of quinolinic acid and neopterin in the cerebrospinal fluid of ASD patients were *lower*, whereas the level of BH4 was *higher* than in healthy controls. This result is compatible with a *decreased* M1 polarization in ASD. The largest post mortem study thus far investigated cortical samples of 47 ASD cases and 57 controls [77]. The transcriptome of each sample was determined, and the data were analyzed for co-expression patterns. The authors made the significant discovery that one of the identified “modules” (clusters of co-expressed genes) was enriched for genes related to the microglial M2 state [77]. Taken together, these data provide support for an increase in microglial M1 polarization in ASD, but also support an increase in M2 polarization. This may either reflect distinct subtypes of ASD, but might also reflect different timely separated phases (e.g., when an infection first induces a defense response [M1] and then a repair [M2A]). 

## 6. Growth Factors from M2A-Polarized Microglia Are Associated with ASD

In accordance with the role of M2A-polarized microglia and macrophages in repair and recovery, these cells produce growth factors that stimulate proliferation of stem cells (reviewed by [58,72]). Upon stimulation by IL4 or IL13, the expression of insulin-like growth factor-1 (IGF1) by microglia [57,65,78] and macrophages [78,79] was strongly increased. IGF1 is an important cytoprotective protein with a relevant function in tissue repair after inflammation [80,81]. Compared to controls, blood levels of IGF1 were significantly increased in male ASD patients who had a large head circumference, and also the head sizes correlated with blood level [82]. The contention that IGF1 is an important factor for head size is also supported by data from genetic studies. Thus, whereas hemizygosity for IGF1R (the receptor for IGF1 and IGF2) is associated with growth failure, individuals who carry *three* copies of the IGF1 receptor present with a pronounced macrocephaly [83]. Importantly, in a candidate gene-study, single nucleotide polymorphisms in the IGF1 gene were associated with Asperger syndrome [84]. 

In addition to IGF1, also BDNF levels may be increased in ASD patients. A meta-analysis of serum and plasma BDNF levels identified 14 studies involving 2707 participants (1131 ASD patients), and noted significantly elevated peripheral BDNF levels in young (*n.b.* not in adult) ASD patients [85]. The study by Schwarz et al. [49] mentioned before reported that BDNF levels in blood were increased in female, but not male Asperger patients. Finally, in a small post mortem study of mainly male adult ASD patients, BDNF-expression in the basal forebrain was threefold higher than in controls [86]. The possibility that BDNF influences brain development is indicated by an MRI study that found that a single nucleotide polymorphism in the BDNF gene affected cortical volume [87]. These findings in patients corroborate the aforementioned evidence that IL4-induced microglia are a source of a number of potent growth factors. 

## 7. Mechanisms by Which Growth Factors May Cause ASD Symptoms

Microglia has a profound influence on neuronal and astroglial function and on brain development [88,89]. One current hypothesis of ASD proposes that glial dysfunction directly contributes to pathophysiology [90]. This hypothesis was formulated mainly on the basis of results from animal studies; for instance, in mice with a genetic deletion of the chemokine receptor CX3CR3 (a receptor that is exclusively expressed on microglia), which led to delayed synaptic pruning, and ultimately resulted in excessive numbers of immature synapses and a diminished performance in cognition tests [11,69,91]. A microglia-specific deletion of BDNF expression resulted in similar deleterious effect on cognitive function [92]. These data clearly indicate that a proper microglia function is important. Decreased BDNF signaling has been thoroughly studied in the context of depression, and numerous transgenic mouse models of impaired BDNF function have been generated (reviewed in [93]). On the other hand, BDNF-*over*expression has rarely been investigated [93]. E.g., in female BDNF-overexpressing mice, working memory was impaired, while they displayed more anxiety and self-grooming, and were more susceptible to seizures than their wild-type littermates [94]. Although this phenotype is reminiscent of ASD, it is unclear whether this study is informative about ASD, because BDNF was overexpressed exclusively in excitatory neurons. Transgenic mice with IGF1-overexpression in neurons or in astrocytes have been generated, too [80]. Such mice exhibited marked increases in brain weight, in number of neurons, astrocytes and oligodendrocytes, as well as increases in myelination and synapse-to-neuron ratio [80,81]. IGF1 stimulates the proliferation of progenitor cells and drives their fate-selection towards neurogenesis [95,96]. A higher IGF1 level could be one of the causes for the increase in neuron numbers that is observed in ASD patients [97] and could represent a partial explanation for the large head circumference [33]. Both BDNF and IGF1 activate the PI3K–Akt pathway. This pathway is important for cell survival and cell growth, whereas apoptosis and macrophage/microglia M1 polarization are suppressed [43,78,93,98,99]. Taken together, the increased expression of BDNF and IGF1 could explain several aspects of ASD. 

## 8. Discussion

The data summarized above indicate that allergy is associated with ASD. Allergy involves an increase in the activation of Th2 lymphocytes, mast cells, innate lymphoid cells and others [45,59,100]. These cell types are known to express and release IL4, IL5 and IL13 [44,59,98,101]. IL4 and IL13 are potent inducers of the M2A phenotype of macrophages and microglia [98]. In fact, not only allergy but also other potential environmental risk factors for ASD, like maternal prenatal stress or prenatal exposure to antidepressant medication, may lead to M2A skewing of phagocytes [25,102,103]. In the M2A-polarized form these cells express and secrete growth factors that activate vascular and neuronal stem cells, improve neurogenesis and neuronal survival, and thereby influence different domains of cognitive function [104]. In certain subtypes of the autism spectrum there is evidence for increases in blood levels of IL4 and/or IL13. There is also evidence for an M2A polarization of microglia, including findings of increased expression of M2A-markers and increased levels of the growth factors VEGF, IGF1 and BDNF. It is therefore conceivable that allergy contributes to the prevalence and severity of ASD via an increased release of growth factors in the brain. The next paragraph describes the putative mechanism of how elevated levels of growth factors could lead to ASD symptoms. 

**Figure 1:**
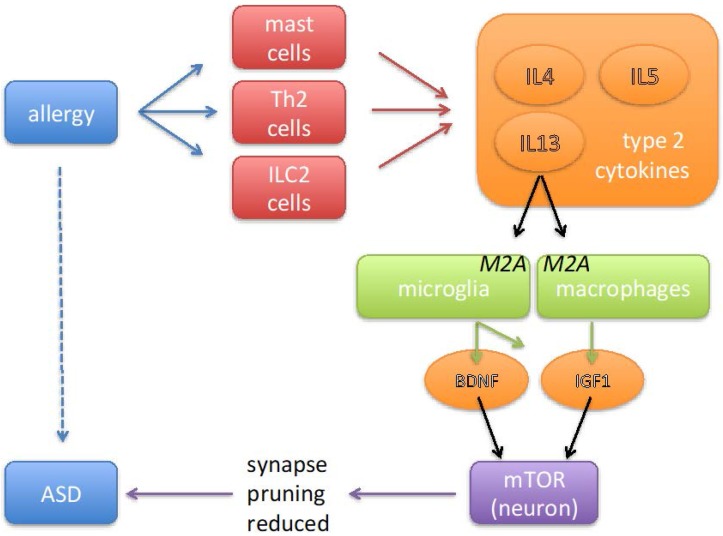
Cartoon summarizing the sequence of events via which allergic disorders may contribute to the pathology of autism spectrum disorders (ASD). Allergy is associated with an activation of mast cells, innate lymphoid cells (ILC) and Th2 T cells. These cells produce type-2 cytokines (IL4, IL5 and IL13), which stimulate microglia and macrophages to adopt a phenotype referred to as ‘alternative activation’ or ‘M2A’. By secreting a variety of growth factors, including brain-derived neurotrophic factor (BDNF) and insulin-like growth factor-1 (IGF1), M2A-polarized macrophages and microglia cells play a physiological role in tissue repair. In neurons these growth factors activate a signal transduction pathway that leads to activation of the enzyme mammalian target of rapamycin (mTOR) and thereby to the inhibition of autophagy. Inhibition of autophagy results in diminished removal of redundant synapses, which in the context of ASD is likely to be undesired, as the results from genetic studies indicate that insufficient synaptic pruning is an effect that is common to several syndromic forms of ASD.

Numerous studies attempted to identify the genetic causes of ASD. Interestingly, such studies frequently detected alterations in genes that are critically important for synapse function [5,6,7,8,9,10]. In particular, Voineagu and Eapen [9] have suggested that immunological processes (either genetically or environmentally induced) contribute to ASD by exerting a negative influence on synapse function. Pruning of non-functional synapses involves an initial interaction between microglia and synapses [11], which is followed by the removal of the redundant synapses via an intra-neuronal autophagic process [12,13]. Stimulation of mTOR leads to an inhibition of autophagy [13], whereas multiple ASD syndromes are caused by mutations in genes that, if intact, function as inhibitors of mTOR. Importantly, via stimulation of the PI3K–Akt–mTOR pathway, both IGF1 and BDNF activate mTOR. In theory, this would hinder the autophagic removal of redundant synapses [80,105]. Following this line of reasoning, allergy-induced M2A polarization, with its increased production of IGF1, BDNF and further growth factors, would contribute to ASD via inhibition of normal pruning of synapses (see Figure 1). A dysfunction of normal pruning may help to explain alterations in cognition and behavior.

It seems that, during the last decades, the prevalence of allergic diseases has increased considerably [106]. As for ASD, this fast increase rather argues against a genetic mechanism, and is probably explainable by environmental factors in combination with an increased awareness [106,107]. Environmental factors that have been proposed include allergens (for instance exposure to house dust mites; [108]), air pollution and passive smoking [108], Western style of living, including an increase in hygiene [21] and industrialized methods for food production [109]. As these environmental factors are not mutually exclusive, it will be difficult to tease out which of these has the greatest impact. In this review, it is argued that allergy represents an environmental risk factor for autism spectrum disorders, which involves a cascade of events including the Th2-cytokines IL4 and IL13, an M2A polarization of microglia, the release of growth factors, and the inhibition of autophagy in neurons (see Figure 1). For this reason, the most logical therapeutic approach would be to reduce the exposure to allergens. The therapeutic response to gluten- or casein-free diets is often strongly advocated by parents [110]; however, results from objective clinical studies are less convincing [111]. Although development of therapies for very young children is particularly challenging, it might prove possible to interfere in the pathological cascade in a manner that the benefit exceeds the risks. For instance, specific antibodies against IL4 or IL13 might be explored in order to prevent an excessive M2A polarization [100]. Alternatively, one could try to interrupt signaling pathways that promote M2A polarization, e.g., by tyrosine kinase inhibitors that block STAT6 phosphorylation [112]. Since mTOR activation seems to be an effect common to several forms of ASD, including the allergy mechanism discussed in the current review, it might be useful to prevent or to inhibit the activation of mTOR [15,113,114]. So in summary, it might very well be possible that allergy represents a treatable risk factor for ASD.
